# Analgesic and Anti-Inflammatory Activities of the Ethanolic Extract of *Artemisia morrisonensis* Hayata in Mice

**DOI:** 10.1155/2012/138954

**Published:** 2012-12-29

**Authors:** Shen-Chieh Chou, Yung-Jia Chiu, Chao-Jung Chen, Ying-Chih Lin, Chung-Hao Wu, Chien-Ti Chao, Ching-Wen Chang, Wen-Huang Peng

**Affiliations:** ^1^Graduate Institute of Ecology and Evolutionary Biology, College of Life Sciences, China Medical University, No. 91 Hsueh-Shih Road, Taichung 40402, Taiwan; ^2^Department of Chinese Pharmaceutical Sciences and Chinese Medicine Resources, College of Pharmacy, China Medical University, No. 91 Hsueh-Shih Road, Taichung 40402, Taiwan; ^3^Graduate Institute of Integrated Medicine, College of Chinese Medicine, China Medical University, No. 91 Hsueh-Shih Road, Taichung 40402, Taiwan; ^4^Proteomics Core Laboratory, Department of Medical Research, China Medical University Hospital, Taichung 40402, Taiwan; ^5^Department of Optometry, Jen-Teh Junior College of Medicine, Nursing and Management, No. 79-9 Sha-Luen-Hu, Xi Zhou Li, Houloung 35664, Taiwan; ^6^School of Pharmacy, College of Pharmacy, China Medical University, No. 91 Hsueh-Shih Road, Taichung 40402, Taiwan; ^7^Department of Forestry, College of Agriculture and Natural Resources, National Chung Hsing University, No. 250 Kuo-Kuang Road, Taichung 40227, Taiwan

## Abstract

The aim of this study was to investigate the possible analgesic and anti-inflammatory mechanisms of the ethanolic extract of *A. morrisonensis* Hayata (AM_EtOH_). Two models were employed for evaluation of the analgesic effects: acetic acid-induced writhing response and formalin-induced paw licking. The results demonstrated that AM_EtOH_ decreased writhing response for both the acetic acid assay and the licking time in the formalin test. The anti-inflammatory effect was evaluated by paw edema of mice induced by **λ**-carrageenan. AM_EtOH_ significantly decreased induced paw edema three to four hours after **λ**-carrageenan injection. Additionally, the results indicated that the anti-inflammatory mechanism of AM_EtOH_ may be due to the declined levels of nitric oxide (NO) and malondialdehyde (MDA) in the edematous paw. Furthermore, AM_EtOH_ decreased the tumor necrosis factor-alpha (TNF-**α**) and interleukin-6 (IL-6) levels, leading to the reduction of prostaglandins and subsequently alleviated edema. Isolation and purification of the AM_EtOH_ extract determined *p*-hydroxyacetophenone to be a major component at 130 mg/g of extract. No mortality was observed in the acute toxicity test given at the dose of 10 g/kg. This study demonstrated the possible mechanisms for the analgesic and anti-inflammatory effects of AM_EtOH_ for mice and provided evidence for the ethnobotanical uses of *A. morrisonensis* in treating inflammatory diseases.

## 1. Introduction

Rheumatoid arthritis (RA) is a chronic, systemic, inflammatory, and immunological disorder causing joint destruction and disability, typically observed in people between the ages of 30 and 50 [1]. This disease affects 0.5–1% of adults and its incidence and prevalence are higher in industrialized countries [2]. Drug treatment for RA includes nonsteroidal anti-inflammatory drugs (NSAIDs), low-dose oral or intra-articular glucocorticoids, disease-modifying anti-rheumatic drugs, biologic response modifiers, and daily calcium/vitamin D [3]. Inflammation is the result of host defense to tissue injuries and/or against pathogenic stimuli; however, persistent or hyperinflammation can lead to tissue damage and eventually to organ failure if not properly controlled. In response to infectious agents or pro-inflammatory stimuli, activated macrophages/monocytes secrete cytokines, growth factors, and inflammatory mediators including interleukin-1 (IL-1), interleukin-6 (IL-6), tumor necrosis factor-alpha (TNF-*α*), nitric oxide (NO), prostaglandin E_2_, and reactive oxygen species (ROS) which will cause inflammation injury [4, 5]. Excessive NO will rapidly combine with superoxide anion (O_2_^−^) to generate peroxynitrite (ONOO^−^), which can lead to pathogenesis by promoting oxidative stress, tissue injury, and cancer. Peroxynitrite is a potent oxidant that reacts with protein, lipids, and DNA, leading to subsequent multistage carcinogenesis [6, 7]. ROS can cause oxidative damage, which in turn can initiate and promote the progression of a variety of chronic diseases, such as cardiovascular diseases, Alzheimer's disease, and diabetes [8]. Therefore, superoxide dismutase (SOD), glutathione (GSH), glutathione peroxidase (GPx), and glutathione reductase (GRd) play crucial roles in ameliorating inflammatory reactions via decreasing oxidative stress and damage through reducing the production of free radicals [9, 10]. Malondialdehyde (MDA), a low-molecular-weight end product formed from decomposition of cell membrane, provides an indicator in the evaluation of the inflammatory process [11]. Also, lipid peroxidation in many disease states is a reliable assessment in oxidative injury. Besides inhibition to PG and NO by suppression of its corresponding synthases, cyclooxygenase-2 (COX-2) and inducible nitric oxide synthases (i-NOS) have demonstrated to be beneficial in the treatment of inflammatory diseases [12]. Natural products from herbal medicine or medicinal plants provide a vast pool of COX-2 and/or NO inhibitors that can possibly be developed into new anti-inflammatory drug leads. The motivation of this research is trying to seek for novel substances of natural origin that will demonstrate effectiveness in anti-inflammatory and analgesic activities with low toxicity. 

*Artemisia* genus (Compositae) is used for treating flu, rheumatoid arthritis, back pain, and headaches among American Indians [13, 14]. *A. morrisonensis *Hayata is an endemic and perennial herb/subshrub found along roadsides or slopes above 2700 m mountains in Taiwan [15]. Traditionally, this plant has been used to treat rheumatoid arthritis, allergic rhinitis, headache, and edema in aboriginals. However, the therapeutic potential, and underlying mechanisms still remain elusive and the active component(s) have not been systematically identified. Phytochemical studies to *Artemisia* genus revealed that monoterpenes, sesquiterpenes, coumarins, and flavonoids with great structural diversity were isolated and identified [16]. The previous studies have indicated that phenolics in medicinal plants possessed anti-inflammatory activities via scavenging ROS and reduced pro-inflammatory cytokines (e.g., TNF-*α*, IL-1*β*, and IL-6). Some research indicated that the content of phenolics increases as the altitude becomes higher due to the protective role against UV [17]. Thus, quantitative analysis by HPLC interfaced to UV-Vis and/or MS is imperative to certain target constituents claimed to be responsible for the treatment of specific diseases.

In this study, the analgesic and anti-inflammatory activities of the ethanolic extract of *A. morrisonensis *Hayata (AM_EtOH_) were evaluated in mice. Analgesic activity was undertaken by acetic acid-induced writhing response and formalin test while the anti-inflammatory activity of AM_EtOH_ was determined by using *λ*-carrageenan-induced mice paw edema model. In order to disclose the mechanisms of anti-inflammatory effect, the activities of TNF-*α*, IL-6, and COX-2 and the levels of NO and MDA in the edematous tissues were assayed, and the activities of antioxidant enzymes (SOD, GPx, and GRd) in the liver were measured. 

## 2. Materials and Methods

### 2.1. Chemicals and Reagents

*λ*-Carrageenan, indomethacin, formic acid (FA), and Griess reagent were purchased from Sigma-Aldrich Chemical Co. (Taipei, Taiwan). Formalin was obtained from Nihon Shiyaku Industries Ltd. (Taipei, Taiwan). SOD, GPx, GRd, and MDA assay kits were purchased from Randox Laboratory Ltd. (Crumlin, UK). IL-6 and TNF-*α* were obtained from Assay Designs Inc. (Michigan, USA). COX-2 antibody was purchased from R&D System. NO assay kit was obtained from Cayman Chemical Co. HPLC-grade methanol and acetonitrile were purchased from Merck (Darmstadt, Germany). All other reagents used were of analytical grade.

### 2.2. Plant Material

*Artemisia morrisonensis *Hayata was collected along the highway 14 A at 2718 m (N24°06′57.8′′ E121°13′52.0′′), Nantou, Taiwan, in June 2011. The plant was identified by Dr. Yen-Hsueh Tseng, Department of Forestry, National Chung Hsing University. A voucher specimen (AM-1-06032011) has been deposited at the Graduate Institute of Ecology and Evolutionary Biology, China Medical University. Dried stems and leaves (260 g) of this plant were sliced into small pieces and extracted with 70% ethanol for four times and passed through filter paper. The combined filtrate was concentrated under reduced pressure to give a dried extract (8.6 g, yield ratio 3.3%). Prior to pharmacological tests, this extract was dissolved in dd H_2_O. 

### 2.3. Chromatographic Separation and Analyses

AM_EtOH_ was dissolved in 250 mL water and partitioned thrice against ethyl acetate with the same volume to give ethyl acetate layer (2.75 g). This layer was chromatographed through silica gel column to give 30 subfractions from which the 22th (222.8 mg) was further purified via Sephadex LH-20 eluted with acetone to yield 122.0 mg of *p*-hydroxyacetophenone. ^1^H-NMR (500 MHz, CDCl_3_): 2.58(s, 3H), 6.95(d, *J* = 8.5 Hz, 2H), 7.90(d, *J* = 8.5 Hz, 2H) and ^13^C-NMR: 26.2(q), 115.6(d), 115.6(d), 129.3(s), 131.2(d), 131.2(d), 161.7(s), 198.9(s). The ESI-MS (negative mode) showed molecular ion at *m/z*: 135 and at 120 and 93 corresponding to the deletion of methyl group and further removal of CO, respectively. The above data was completely consistent with those of *p*-hydroxyacetophenone reported and confirmed by its structure. The HPLC system consisted of a Hitachi L-2130 detected by UV-Vis detector L-2420. Chromatographic separation was performed on NUCLEODUR C-18 HTec (4.6 mm × 250 mm ID, 5 *μ*m) with an auto-sampler L-2200. The mobile phase consisted of a mixture of water (A) and acetonitrile (B) using a gradient program set up as follows: 0 min: 5% B, 5 min: 30% B, 30 min: 95% B, and 50 min: 5% B. The flow rate was 1.0 mL/min and the detection wavelength was at 315 nm. The LC-MS was performed on Phenomenex Prodigy ODS(2), 5 m, 532610-1, 2.0 mm × 150 mm with flow rate of 250 *μ*L/min coupled to Bruker HCT Ultra Ion Trap MS spectrometer. The mobile phase A was H_2_O (+0.1% FA) + 5% ACN (+0.1% FA) and mobile phase B was ACN (+0.1% FA). The gradient program was set as follows: 0 min: 10% B, 15 min: 60% B, 17–20 min: 90% B, and 21–25 min: 10% B. The identity of *p*-hydroxyacetophenone was determined by retention time and molecular and fragment ions found in LC-MS. Quantitative analysis was made by the establishment of calibration curve with peak areas under curves in the standard addition method. The concentration of AM_EtOH_ was prepared as 5 mg/mL and spiked with 0, 0.5, 1, 2, and 5 mg/mL of *p*-hydroxyacetophenone and the amount of this component was determined as 0.13 ± 0.15 g/g extract (mean ± RSD) with *R* ^2^ > 0.99. 

### 2.4. Experimental Animals

Male ICR mice (20–23 g each for analgesic test and 32–35 g each for anti-inflammatory test) were provided by BioLASCO Taiwan Co., Ltd. The mice were kept in the animal center of the China Medical University at a controlled temperature of 22 ± 1°C, relative humidity 55 ± 5%, and with 12 h light/12 h dark cycles for 1 week before the experiment. Animals were given with rodent diet and clean water *ad libitum*. All studies were conducted in accordance with the National Institutes of Health (NIH) Guide for the Care and Use of Laboratory Animals. All tests were conducted under the guidelines of the International Association for the Study of Pain [18]. The experimental protocol was approved by the Committee on Animal Research, China Medical University. Ether was used to anesthetize the animals before sacrificing them.

### 2.5. Acute Toxicity Study

The acute toxicity test in mice was performed according to the method of Liao et al. [19]. Male ICR mice (22–25 g) were randomly divided into three groups with 10 mice in each and the mice were administered orally with AM_EtOH_ (10 g/kg). The experimental mice were given with forage and water *ad libitum* and were kept under regular observation for any mortality or behavioral changes within 14 days.

### 2.6. Acetic Acid-Induced Writhing Response

The writhing test in mice was conducted by the method of Koster et al. [20]. Male ICR mice (*n* = 10) were fasted for 24 h before the experiment with free access to water. Acetic acid (1.0%) was injected intraperitoneally (i.p., *v*/*v*, 0.1 mL/10 g body weight) to induce writhing. AM_EtOH_ (20, 100, and 500 mg/kg) was orally administered (p.o.) to each group of mice 60 min before acetic acid injection. Indomethacin (10 mg/kg) was administered intraperitoneally as a positive control 30 min before the injection of acetic acid. The number of muscular contractions was counted 5 min after the injection of acetic acid and lasted for over 10 min. The recorded data represented the total number of writhes observed.

### 2.7. Formalin Test

The formalin test was conducted based on the method of Tjølsen et al. [21]. Male ICR mice (*n* = 10) were fasted for 24 h before the experiment with free access to water. Twenty microliter of 5% formalin in saline was injected subcutaneously into the right hind paw of each mouse. AM_EtOH_ (20, 100, and 500 mg/kg) were orally administered to the animals 60 min prior to the formalin injection. The same volume of distilled water was orally administered as the vehicle control. Indomethacin (10 mg/kg, i.p.) was administered 30 min before formalin treatment. These mice were individually placed in a transparent Plexiglas cage (25 *× *15 *× *15 cm). The time spent in seconds on licking and biting the injected paw was recorded in both of the early phase (0–5 min) and late phase (20–30 min) after the formalin injection as neurogenic and inflammatory pain, respectively. 

### 2.8. *λ*-Carrageenan-Induced Mice Paw Edema

The test was conducted according to the method of Vinegar et al. with some modification [22]. Male ICR mice (*n* = 10) were fasted for 24 h before the experiment with free access to water. Fifty microliter of 1%  *λ*-carrageenan suspended in normal saline solution (0.9% w/v NaCl) was injected into the plantar side of right hind paw and the paw volume was measured by a plethysmometer at the 1st, 2nd, 3rd, and 4th hour after the injection. The degree of swelling was evaluated by the delta volume (*a* − *b*) where “*a*” stands for the volume of the right hind paw after the chemical treatment and “*b*” being the volume before the treatment. AM_EtOH_ (20, 100, and 500 mg/kg) and normal saline solution were administered orally after 120 min for experimental and vehicle control after *λ*-carrageenan injection, respectively. Indomethacin (10 mg/kg, i.p.) was administered 150 min after the *λ*-carrageenan injection for positive control. In the secondary experiment, another set of mice were orally administered with normal saline, AM_EtOH_, and indomethacin with the same condition described previously. The right hind paws and liver tissues of the mice were taken at the fourth hour after *λ*-carrageenan injection. The paw tissue was rinsed and immediately placed in ice-cold normal saline four times its volume before homogenization at 4°C. Subsequently, the homogenate was centrifuged at 12,000 rpm for 5 min and the supernatant was obtained and stored at −20°C for MDA, COX-2, NO, TNF-*α*, and IL-6 analyses. The whole liver tissue was rinsed and placed in ice-cold normal saline with equal volume before homogenization at 4°C. The homogenate was centrifuged at 12,000 rpm for 5 min and the supernatant was obtained and stored at −20°C for antioxidant enzymes (superoxide dismutase, glutathione peroxidase, and glutathione reductase) activity analysis. 

### 2.9. COX-2, TNF-*α*, and IL-6 Assay

COX-2, TNF-**α**, and IL-6 were measured by a quantitative sandwich enzyme immunoassay technique [23]. The capture antibodies of COX-2, TNF-**α**, and IL-6 were seeded to each well of a 96-well plate overnight. In the next day, a second set of the biotinylated antibody was incubated with sample tissues or standard antigens in the plate before streptavidin-HRP was finally added. COX-2 and TNF-**α** were measured at 450 nm to determine their amount. The COX-2 activity was expressed as U/mL per protein (U/mL/mg protein) whereas TNF-**α** was represented as picogram per milligram protein (pg/mg). The absorption of IL-6 was measured at 405 nm and was represented as pg/mg.

### 2.10. NO Assay

NO was measured based on the method of Moshage and Green et al. [24, 25]. Nitrate was converted to nitrite utilizing nitrate reductase. Nitrite subsequently reacted with sulfanilic acid to produce diazonium ion and reacted with N-(1-naphthyl)ethylenediamine to give chromophoric azo derivative (purplish red), which could be recorded at 540 nm. Values obtained by this procedure represented the sum of nitrite and nitrate and represented as M.

### 2.11. MDA Assay

The injection of *λ*-carrageenan will induce the production of MDA, which is evaluated by the 2-thiobarbituric acid reacting substance (TBARS) method [26]. MDA can react with thiobarbituric acid (TBA) under the acidic and high-temperature conditions. MDA and TBA then formed a red-complex TBARS, which can be measured colorimetrically. The absorbance of TBARS was determined by measurement of 532 nm. MDA levels were expressed as nmole MDA/mg protein. 

### 2.12. Measurement of Antioxidant Enzymes

SOD was measured following the method of Woolliams et al. [27]. Xanthine and xanthine oxidase generated superoxide radicals reacting with 2-(4-iodophenyl)-3-(4-nitrophenyl)-5-phenyltetrazolium chloride (INT) to form a red formazan dye whose color was recorded at 540 nm to determine its amount. previous supernatant from liver tissue (20 *μ*L) was added to 120 *μ*L of 0.1 M phosphate buffer (CAPS 40 mmol/L, EDTA 0.94 mmol/L, pH 7.0) and mixed well. An aliquot of 5 *μ*L mixture was added to 340 *μ*L of mixed substrate (xanthine 0.05 mmol/L, INT 0.025 mmol/L) followed by adding xanthine oxidase to record at 505 nm, 37°C at intervals of 30 sec for six times on a Hitachi U 2000 spectrophotometer. The enzyme activity was represented as the amount that inhibited the oxidation of INT by 50% from which is equal to 1 unit (U) and expressed as U/mg protein. GPx was measured according to the method of Ceballos-Picot et al. by detecting the contents of GRd and NADPH [28]. Oxidation of NADPH into NADP^+^ is accompanied by a decrease in absorbance recorded at 340 nm. GRd was measured while detecting the decrease of glutathione (GSSG) in the presence of NADPH [29]. 

### 2.13. Statistical Analysis

All data represented the mean ± SE (*n* = 10). Statistical analyses were performed with SPSS software and were calculated using one-way ANOVA followed by Scheffe's multiple range tests. The criterion for statistical significance was determined as *P* < 0.05. 

## 3. Results

### 3.1. Chromatographic Analyses of AM_EtOH_

**Figure 1:**
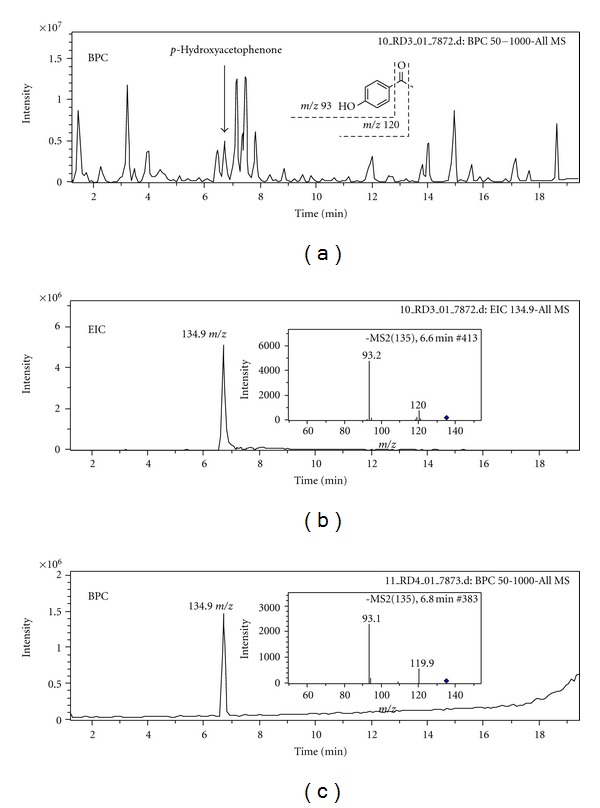
(a) Base peak chromatogram (BPC) from LC-MS of AM_EtOH_. (b) Extracted ion chromatogram (EIC) from *m*/*z* 134.9 of AM_EtOH_; fragment ions were shown as insert. (c) BPC of authentic *p*-hydroxyacetophenone; fragment ions were shown as insert. All chromatograms were detected in the negative ion mode.

**Figure 2:**
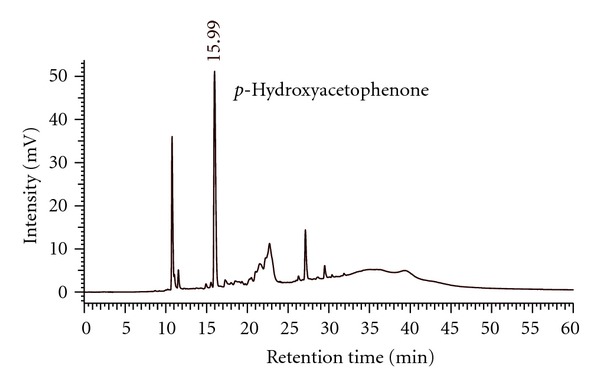
HPLC chromatogram of AM_EtOH_ detected at 315 nm; *p*-hydroxyacetophenone is identified at RT = 15.99 min.

LC-MS fingerprint profile was established as base peak chromatogram (BPC) for AM_EtOH_ (Figure 1(a)) and *p*-hydroxyacetophenone was identified with retention time (RT) at 6.6 min. Extracted ion chromatogram (EIC) was shown in Figure 1(b) as *m*/*z* at 134.9 and was selected in negative ion mode. BPC for authentic *p*-hydroxyacetophenone was shown in Figure 1(c) and was consistent with RT, molecular and fragment ions. The chromatogram detected at 315 nm was shown in Figure 2 with RT at 15.99 min and was used for quantitative analysis. 

### 3.2. Acute Toxicity Study

Acute toxicity of AM_EtOH_ was evaluated in mice at the doses of 10 g/kg. After 14 days of oral administration, AM_EtOH_ did not cause any behavioral changes and no mortality was observed. Therefore, the LD_50_ of AM_EtOH_ was concluded to be greater than 10 g/kg in mice, indicating that it was practically nonacute toxic.

### 3.3. Acetic Acid-Induced Writhing Response

**Figure 3:**
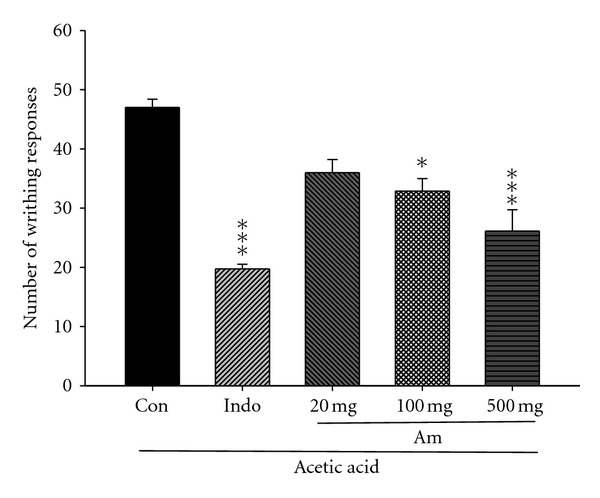
Analgesic effects of ethanol extract of AM_EtOH_ and indomethacin (Indo) on acetic acid-induced writhing response in mice. Each value represents as mean ± SEM (*n* = 10), *P < 0.05, ***P < 0.001 as compared with the control (Con) group. (One-way ANOVA followed by Scheffe's multiple range test.)

Figure 3 shows that acetic acid-induced writhing responses in mice serve as an indication of analgesic activities exerted by AM_EtOH_. Intraperitoneal injection of acetic acid produced 47.0 ± 1.4 writhes in the control group. The writhing response was significantly reduced by treatment with indomethacin (10 mg/kg) or AM_EtOH_ at 100 and 500 mg/kg with *P* being less than 0.05 and 0.001, respectively.

### 3.4. Formalin Test

**Figure 4:**
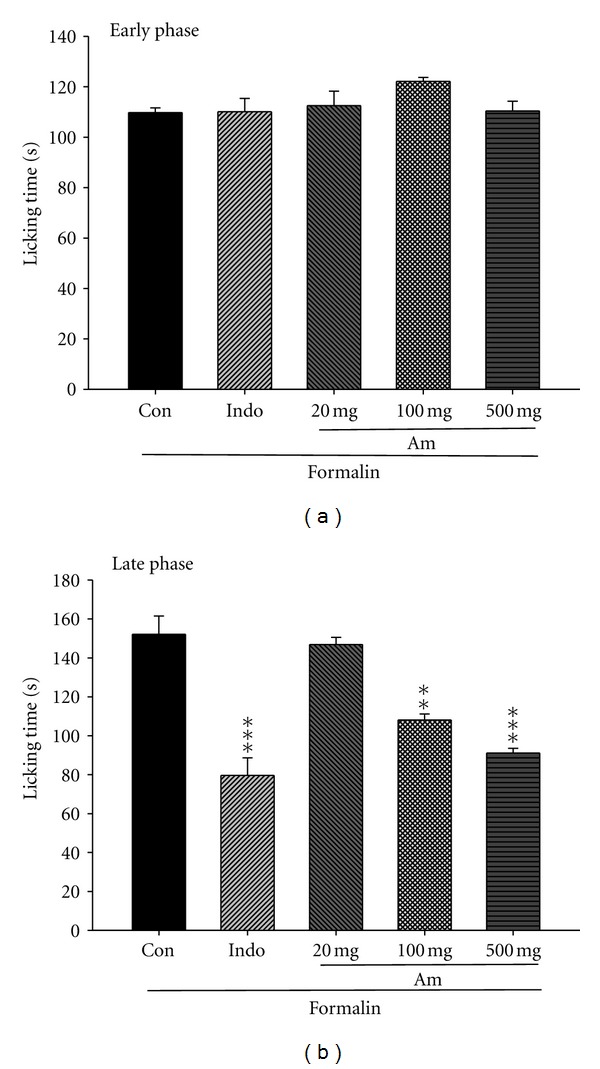
Analgesic effects of AM_EtOH_ and indomethacin (Indo) on the (a) early phase and (b) late phase in formalin test in mice. Each value represents as mean ± S.E.M. (*n* = 10), **P < 0.01, ***P < 0.001 as compared with the control (Con) group. (One-way ANOVA followed by Scheffe's multiple range test.)

In the early phase, AM_EtOH_ (20, 100, and 500 mg/kg) and indomethacin (10 mg/kg) did not show any significant changes compared with the control group (Figure 4(a)). In the late phase, licking and biting responses induced by subcutaneous injection of formalin have lasted for 146.8 ± 3.7 s in the control group. As shown in Figure 4(b), the time decreased significantly with indomethacin (10 mg/kg) or by treatment with AM_EtOH_ at 100 and 500 mg/kg with *P* being less than 0.05 and 0.001, respectively.

### 3.5. Effect of AM_EtOH_ on *λ*-Carrageenan-Induced Mice Paw Edema

**Figure 5:**
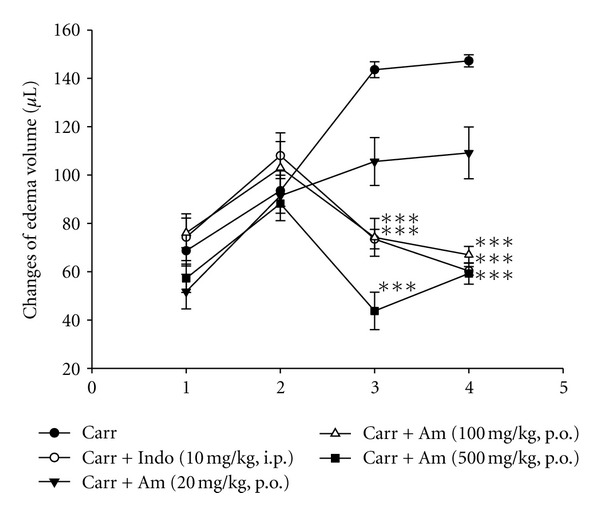
Effects of AM_EtOH_ and indomethacin (Indo) on hind paw edema induced by *λ*-carrageenan in mice. Each value represents as mean ± S.E.M. (*n* = 10), ***P < 0.001 as compared with the *λ*-carrageenan (Carr) group. (One-way ANOVA followed by Scheffe's multiple range test.)

As shown in Figure 5, the volume of mouse paw increased as edema developed and served as an indication of inflammatory activity after injection of *λ*-carrageenan. AM_EtOH_ (100 and 500 mg/kg) and indomethacin (10 mg/kg) significantly decreased paw edema at the 3rd and 4th h after the injection. AM_EtOH_ at the concentration of 100 and 500 mg/kg demonstrated almost as equal activity with inhibition as indomethacin (10 mg/kg) with *P* being less than 0.001.

### 3.6. Effect of AM_EtOH_ on COX-2 Concentration

**Figure 6:**
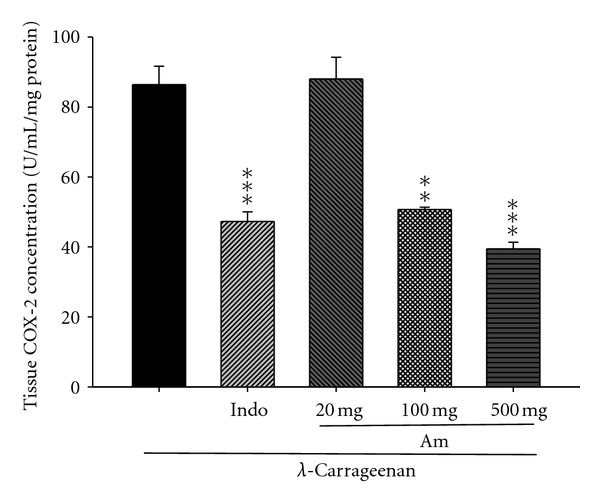
Effects of AM_EtOH_ and indomethacin (Indo) on the tissue COX-2 concentration of edema paw in mice. Each value represents as mean ± S.E.M. (*n* = 10), **P < 0.01, ***P < 0.001 as compared with the Carr group. (One-way ANOVA followed by Scheffe's multiple range test.)

In Figure 6, COX-2 concentration dramatically increased in the *λ*-carrageenan group (U/mL/mg protein). After the treatment with indomethacin (10 mg/kg) or AM_EtOH_ at 100 and 500 mg/kg, significant inhibition was observed in the COX-2 concentration with *P* being less than 0.01 and 0.001, respectively.

### 3.7. Effects of AM_EtOH_ on TNF-*α* and IL-6

**Figure 7:**
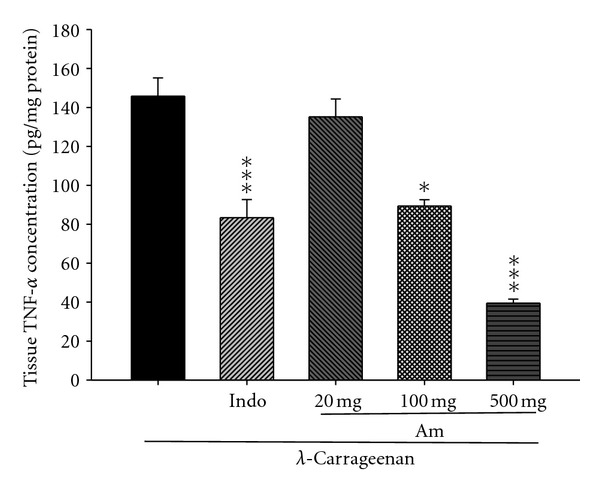
Effects of AM_EtOH_ and indomethacin (Indo) on the tissue TNF-*α* concentration of edema paw in mice. Each value represents as means ± SEM (*n* = 10), *P < 0.05, ***P < 0.001 as compared with the Carr group. (One-way ANOVA followed by Scheffe's multiple range test.)

**Figure 8:**
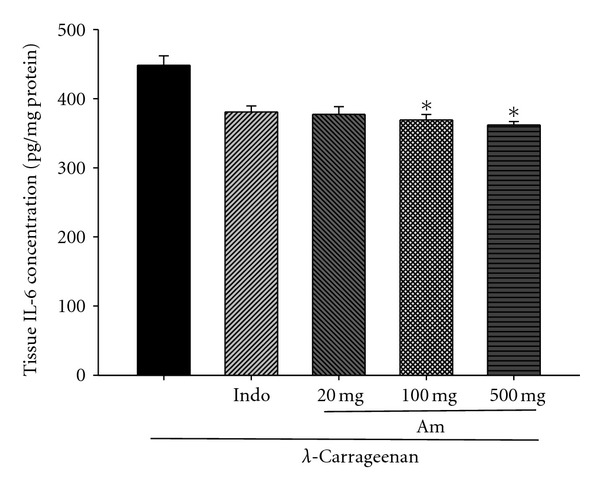
Effects of AM_EtOH_ and indomethacin (Indo) on the tissue IL-6 concentration of edema paw in mice. Each value represents as means ± SEM (*n* = 10), *P < 0.05 as compared with the Carr group. (One-way ANOVA followed by Scheffe's multiple range test.)

As shown in Figures 7 and 8, TNF-**α** and IL-6 levels in *λ*-carrageenan-induced edema paws remarkably raised. Treatment with indomethacin (10 mg/kg) or AM_EtOH_ at 100 and 500 mg/kg significantly suppressed the concentration of TNF-*α* with *P* being less than 0.05 and 0.001, respectively (Figure 7). Similarly, IL-6 levels were significantly lowered by AM_EtOH_ (100 and 500 mg/kg) 3rd h after injection or indomethacin (10 mg/kg) with *P* being less than 0.05 (Figure 8). 

### 3.8. Effect of AM_EtOH_ on NO Concentration

**Figure 9:**
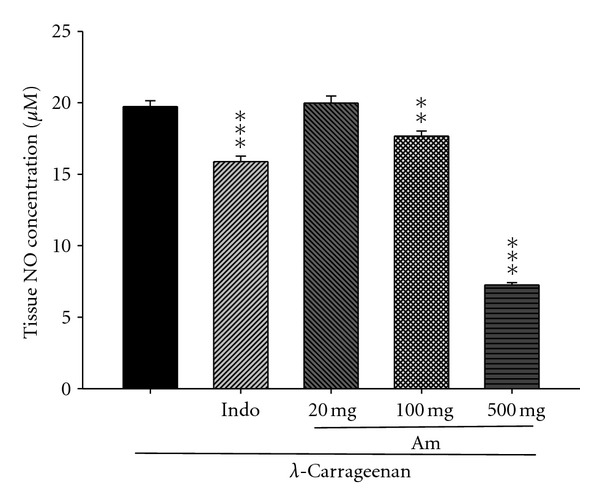
Effects of AM_EtOH_ and indomethacin (Indo) on the tissue NO concentration of edema paw in mice. Each value represents as mean ± S.E.M. (*n* = 10), **P < 0.01, ***P < 0.001 as compared with the Carr group. (One-way ANOVA followed by Scheffe's multiple range test.)

In Figure 9, NO concentration dramatically increased in the *λ*-carrageenan group (*μ*M). After the treatment with indomethacin (10 mg/kg) or AM_EtOH_ at 100 and 500 mg/kg, significant inhibition was observed in the NO concentration with *P* being less than 0.01 and 0.001, respectively.

### 3.9. Effect of AM_EtOH_ on MDA Level

**Figure 10:**
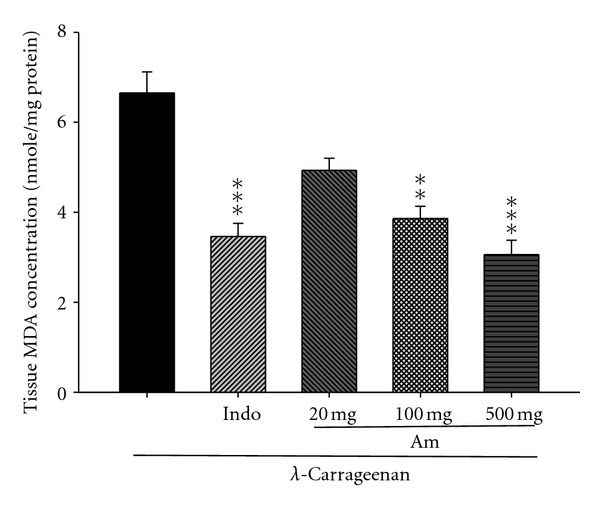
Effects of AM_EtOH_ and indomethacin (Indo) on the tissue MDA concentration of edema paw in mice. Each value represents as mean ± S.E.M. (*n* = 10), **P < 0.01, ***P < 0.001 as compared with the Carr group. (One-way ANOVA followed by Scheffe's multiple range test.)

In Figure 10, MDA level dramatically increased in the *λ*-carrageenan group (nmole/mg protein). After the treatment with indomethacin (10 mg/kg) or AM_EtOH_ at 100 and 500 mg/kg, significant inhibition was observed in the concentration of MDA with *P* being less than 0.01 and 0.001, respectively.

### 3.10. Effect of AM_EtOH_ on the Activity of Superoxide Dismutase (SOD)

**Figure 11:**
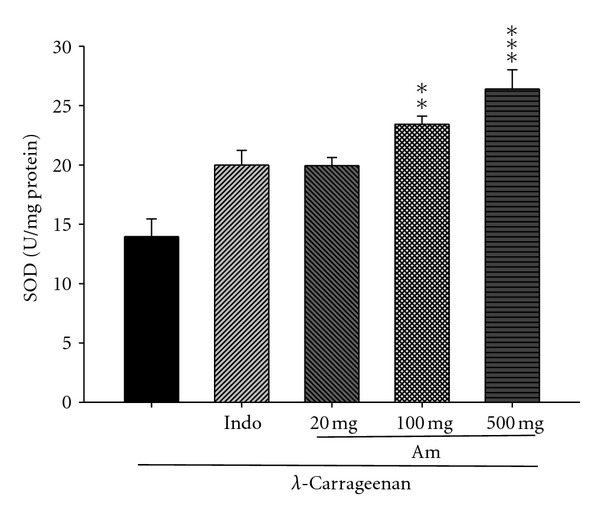
Effects of AM_EtOH_ and indomethacin (Indo) on the liver SOD activities in mice. Each value represents as mean ± SEM (*n* = 10), **P < 0.01, ***P < 0.001 as compared with the Carr group. (One-way ANOVA followed by Scheffe's multiple range test.)

In Figure 11, SOD activity increased by treatment with indomethacin (10 mg/kg) or AM_EtOH_ at 100 and 500 mg/kg compared to that of *λ*-carrageenan group with *P* being less than 0.01 and 0.001, respectively.

### 3.11. Effect of AM_EtOH_ on the Activity of Glutathione Peroxidase (GPx)

**Figure 12:**
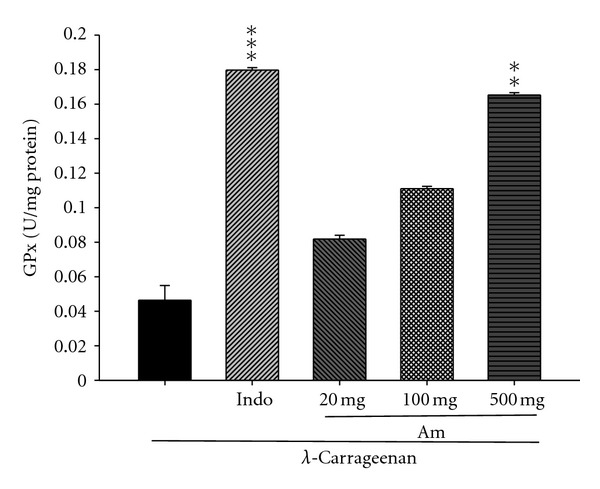
Effects of AM_EtOH_ and indomethacin (Indo) on the liver GPx activities in mice. Each value represents as mean ± SEM (*n* = 10), **P < 0.01, ***P < 0.001 as compared with the Carr group. (One-way ANOVA followed by Scheffe's multiple range test.)

In Figure 12, GPx activity increased by treatment with indomethacin (10 mg/kg) or AM_EtOH_ at 500 mg/kg compared to that of *λ*-carrageenan group with *P* being less than 0.01.

### 3.12. Effect of AM_EtOH_ on the Activity of Glutathione Reductase (GRd)

**Figure 13:**
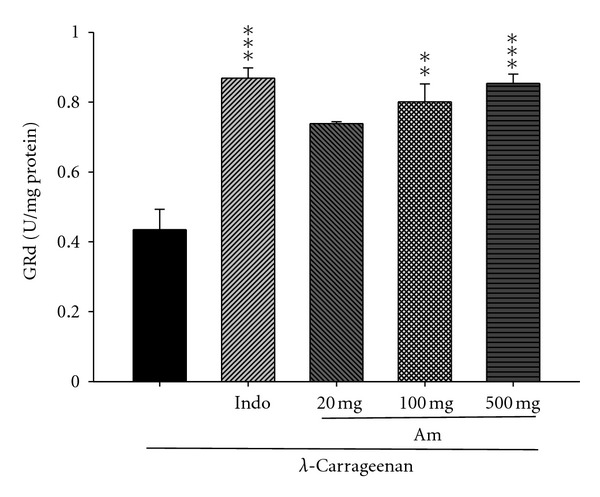
Effects of AM_EtOH_ and indomethacin (Indo) on the liver GRd activities in mice. Each value represents as mean ± S.E.M. (*n* = 10), **P < 0.01, ***P < 0.001 as compared with the Carr group. (One-way ANOVA followed by Scheffe's multiple range test.)

In Figure 13, GRd activity increased by treatment with indomethacin (10 mg/kg) or AM_EtOH_ at 100 and 500 mg/kg compared to that of *λ*-carrageenan group with *P* being less than 0.01 and 0.001, respectively.

## 4. Discussion

Compositaceous plants are well known for their anti-inflammatory and hepatoprotective activities as well as dietary supplements for chemofpreventive purposes in a certain cancer based on ethnopharmacological evidences [30]. *Artemisia* is a genus of about 400 species found in temperate regions worldwide. The chemical constituents from the well-known *A. indica* are mainly volatile oils, coumarins, and flavonoids; however, the phytochemicals in *A. morrisonensis* Hayata are still unclear [16, 31]. We, therefore, embarked on the investigation into active ingredients and possible mechanisms of analgesic and anti-inflammatory effects of *A. morrisonensis* Hayata.

Two animal models including acetic acid-induced writhing response and formalin test were employed to evaluate the analgesic effects of AM_EtOH_. The nociceptive responses induced by acetic acid had been demonstrated to be the involvement of eicosanoids and sympathomimetic amines. The analgesic activity of acetic acid-induced writhing model might attribute to the release of TNF-*α*, interleukin-1 (IL-1), and interleukin-8 (IL-8) by resident peritoneal macrophages and mast cell in mice [32]. In this study, AM_EtOH_ (100 and 500 mg/kg) possessed anti-nociceptive effects to relieve abdominal writhes induced by acetic acid in mice. Formalin test involved a biphasic pain response including a transient early phase followed by a tonic late phase. The early phase was mediated by peripheral nociceptive stimulation caused by formalin while the tonic late phase was due to inflammatory response. However, recent research indicated that the pain response in the late phase depended on the prolonged presence of the early phase, therefore, being interdependent [33]. The present study showed that the treatment of AM_EtOH_ (100 and 500 mg/kg) diminished the nociceptive response in the second phase induced in the formalin test. The results indicated that the analgesic effect exerted by AM_EtOH_ might result from its anti-inflammatory activity. *λ*-Carrageenan-induced paw edema in mice is a biphasic development as well in the evaluation of anti-inflammatory agents *in vivo *[22]. Histamine, bradykinin, 5-hydroxytryptamine (5-HT), and platelet activating factor (PAF) were released in the first phase occurred from 1 to 4 h after injection. Agents derived from the injured tissues stimulated the release of TNF-*α* which further stimulated the release of IL-1, IL-6, and IL-8. IL-6 and IL-8 in turn increased the cyclooxygenase which catalyzed the conversion of arachidonic acid to prostaglandins and thromboxanes. The mRNA expression of inducible COX-2 was markedly increased at the site of *λ*-carrageenan-induced paw edema [34]. TNF-*α* stimulated the release of cytokine-induced neutrophil chemoattractant 1 (CINC-1) associated with IL-8 to mediate the sympathetic pain by stimulating the release of sympathetic amines [35]. Neutrophil infiltration and activation also contributed to this inflammatory response by producing oxygen-derived free radicals, that is, superoxide anion (O_2_^−^) and hydroxyl radicals [36]. Four to 10 hours after *λ*-carrageenan injection, NO produced from i-NOS was involved in the maintenance of inflammatory responses, including the increase in vascular permeability and edema through changes in local blood flow. Nitric oxide reacted with superoxide anion to become peroxynitrite (ONOO^−^), which is proposed to play a major pathogenic role in the inflammatory process especially in macrophage-like cells. In this study, AM_EtOH_ showed significant anti-inflammatory effect in *λ*-carrageenan-induced mice paw edema from the 3rd to the 4th hour in a dose-dependent manner. Furthermore, the levels of TNF-*α* and IL-6 were also decreased by treating with AM_EtOH_ and indomethacin. Therefore, the plausible anti-inflammatory mechanisms of AM_EtOH_ might be associated with the inhibition of TNF-*α* and IL-6. In acute inflammatory responses, reactive oxygen species (ROS), including hydrogen peroxide, hydroxyl radical, and superoxide anion in neutrophil all play pivotal roles, hence leading to DNA, lipid, and protein damage [37]. MDA is a reactive aldehyde that serves as a biomarker in the evaluation of oxidative stress and inflammatory process. Antioxidant defense systems scavenge and minimize the formation of ROS through enzymes, such as SOD, GPx, and GRd [38]. SOD catalyzes the conversion of superoxide anion to hydrogen peroxide followed by catalases to water and oxygen. GPx removes hydrogen peroxide to water and oxygen, in the meantime, giving oxidized glutathione (GSSH), which subsequently regenerates via GRd with NADPH as a source of hydrogen. Therefore, the increment of SOD, GPx, and GRd will diminish ROS and serves as the assessment of the degree of inflammation. Cytokine is a group of polypeptides synthesized by the host in response to immunity and inflammatory responses. For instance, IL-1, TNF-*α*, and IL-6 have been reported in implication in the inflammatory response. IL-1 and TNF-*α* also regulate the expression of i-NOS and COX-2 and subsequently modulate NO and PGE_2_ [39]. Efforts to attenuate these pleiotropic inflammatory mediators have been attracting much attention in the development of new anti-inflammatory drugs. The major component from AM_EtOH_ is *p*-hydroxyacetophenone whose structure was totally consistent with the literature data in NMR spectroscopy [40]. Quantitative analysis was performed by HPLC detected at 315 nm with standard addition method [41]. The major ingredient in *Lonicera japonica* is chlorogenic acid whose anti-inflammatory activity was confirmed [42, 43]. Chromatogram showed that chlorogenic acid was identified at RT of 3.9 min. However, the amount was tiny in our observation though it had been found from *A. scopariae *[44]. One study showed that *p*-hydroxyacetophenone produced protective effects in *λ*-carrageenan-induced paw edema in mouse at 1–3 h after the injection [45]. Quantitative study to *p*-hydroxyacetophenone had been reported from *A. scopariae* by capillary electrophoresis and determined to be 6.732–8.795 mg/g [44]. In this study, the major component in AM_EtOH_ was *p*-hydroxyacetophenone and the amount was determined to be 130 mg/g.

This study demonstrated that AM_EtOH_ possessed analgesic activity against nociceptive responses induced by the intraperitoneal injection of acetic acid at concentration of 100 and 500 mg/kg. The paw licking time was significantly reduced in the late phase by intraplantar injection of formalin at 100 and 500 mg/kg. AM_EtOH_ declined the paw edema volume at dose of 100 and 500 mg/kg induced by *λ*-carrageenan. Two possible pathways were proposed for the anti-inflammatory effect exerted by AM_EtOH_. The first was the reduction of the concentration of TNF-*α* and IL-6, not only for the suppression of COX-2 leading to a decrease in PG released but also for the decrease of NO concentration to prevent edema. The second was by increasing the activity of anti-oxidant enzymes (SOD, GPx, and GRd) in the liver, in which the free radicals were scavenged and the level of MDA was declined. The analgesic effect was found to be related to its anti-inflammatory activity and served as a possible rationale on AM_EtOH_ in traditional medicine.
